# Do female and male patients with cervical dystonia respond differently to long-term botulinum neurotoxin therapy?

**DOI:** 10.3389/fneur.2025.1553989

**Published:** 2025-06-17

**Authors:** Harald Hefter, Sara Samadzadeh, Marek Moll

**Affiliations:** ^1^Departments of Neurology, University of Düsseldorf, Moorenstrasse, Germany; ^2^Experimental and Clinical Research Center, Charité–Universitätsmedizin Berlin, Freie Universität Berlin and Humboldt-Unverstät zu Berlin, Berlin, Germany; ^3^Department of Regional Health Research and Molecular Medicine, University of Southern Denmark, Odense, Denmark; ^4^Department of Neurology, Slagelse Hospital, Slagelse, Denmark

**Keywords:** cervical dystonia, botulinum neurotoxin, long-term outcome, females, males, antibody formation

## Abstract

**Background:**

Idiopathic cervical dystonia (CD) affects more female than male patients. This sex-based influence on the clinical manifestation of CD may also affect outcomes after long-term BoNT therapy.

**Methods:**

To analyze the potential differential influence of sex on the efficacy of BoNT therapy, a cross-sectional study was conducted with 135 female and 85 male patients with idiopathic cervical dystonia. Demographic and treatment-related data were extracted from patient charts. The 24-item Cervical Dystonia Questionnaire (CDQ24), patients’ self-assessment of CD severity (PAS), and the TSUI score were used as outcome measures. On the day of recruitment, blood samples were collected to analyze neutralizing antibody (NAB) formation using an ELISA, with confirmation by the mouse hemidiaphragm assay (MHDA) test.

**Results:**

Male patients had significantly (*p* < 0.02) lower mean age and age at symptom onset and received significantly (*p* < 0.02) higher BoNT doses per session. Female patients had significantly worse PAS and CDQ24 scores (*p* < 0.006), though the TSUI score showed no significant difference (*p* = 0.19). Despite receiving lower BoNT doses, female patients exhibited a significantly (*p* < 0.006) higher risk of NAB induction.

**Conclusion:**

This reanalysis of previously published data reveals that female and male patients in this cohort were treated and responded differently to long-term BoNT therapy, a discrepancy that remained unnoticed by their treating physicians over more than 10 years of treatment.

## 1 Introduction

Cervical dystonia (CD) affects approximately 100 out of 1 million people in Europe (1). Various epidemiological studies (2–4) across the world reveal a large variation in CD prevalence, ranging from 1:50,000 in Japan (5) to 1:250 in some regions of the United States (US) (6). Ethnic differences in CD prevalence (7, 8) indicate possible genetic influences on CD manifestation.

This finding is in line with the high variability of the female-to-male ratio, ranging from 4.5:1 (9) to 0.3:1 (10). In the abstract of an epidemiological survey, a 2:1 female-to-male ratio was mentioned, while the overall mean female-to-male ratio across 16 articles was 1.7:1 (1). These findings support a possible genetic, or even x-chromosomal influence, on the pathogenesis of CD.

Therefore, it may very well be the case that female and male patients respond differently to the therapy of CD. The therapy of choice for CD is repetitive intramuscular injections of botulinum neurotoxin type A or B (BoNT/A or BoNT/B), which must be applied repetitively to yield a stable plateau of improvement (11). BoNT injection therapy has proven to be a safe and effective long-term treatment without serious side effects. Therefore, it has gained level A recommendations from both the American Academy of Neurology (12) and the British Royal College of Physicians (13) for the treatment of CD.

Sensitivity to BoNT may vary from patient to patient (14). Therefore, at the onset of BoNT therapy, the dose must be adapted to the severity of CD, the patient’s side effect profile, and the patient’s response. After 2 years of continuous treatment, a stable plateau of improvement is typically reached, with little variation in the dose thereafter (11, 14). Thus, the dose per session used in the long-term BoNT treatment is the result of a close interaction between the treating physician and the patient to optimize outcomes and reduce side effects. Therefore, the dose per session is a relevant parameter that may reflect differences in the therapy of female and male patients.

The phenomenology of CD in female and male patients is similar; however, body weight (BW) and total mass of muscles are significantly different. Therefore, the question arises whether these differences are taken into account in the disease management and clinical practice of CD. In the BoNT treatment of children with cerebral palsy, it is common sense to adapt the dose to body weight (U/kg BW) (15–17). In adult patients with CD, such recommendations do not exist.

Furthermore, little is known about whether antibody formation is the same in female and male patients and whether a treating physician reacts the same way when a female or male patient starts to develop partial secondary treatment failure (pSTF) and induction of neutralizing antibodies (NABs) has to be suspected.

Therefore, the present re-analysis of a cross-sectional study, originally published in 2015 (11), was conducted to compare long-term outcomes in female and male CD patients after continuous treatment with BoNT over 2 to 22 years, which were measured using the quality of life questionnaire (CDQ24 (18)), patients’ self-assessment of BoNT therapy (PAS), and physicians’ evaluation of the remaining severity of CD using the TSUI score (19), as well as the prevalence of NABs.

## 2 Methods

### 2.1 Patients and design of the study

This study was performed in accordance with the ethical principles outlined in the Declaration of Helsinki. It is a retrospective re-analysis of a cross-sectional study published in 2015 (11). Therefore, no new vote by the local ethics committee was necessary.

For the cross-sectional study, data from 220 still-responding CD patients were collected between 1 January 2010 and 30 April 2010. The inclusion criteria were as follows: (i) complete documentation of all previous injections, (ii) observation of the last 10 BoNT injection cycles in the BoNT outpatient clinic of the University of Düsseldorf (Germany), (iii) no interruption of BoNT treatment for longer than 4 months during the last 3 years before recruitment, (iv) confirmation of a positive treatment effect by the treating physician and patient, and (v) written informed consent. The exclusion criteria were as follows: (i) patient under medical care and (ii) CD patients receiving BoNT treatment for an additional indication (e.g., blepharospasm) with a dose >10% more than the CD dose.

In the first step, patients completed the CDQ24 questionnaire (18) in a nearby quiet office. Then, they underwent a detailed clinical investigation, including the measurement of body weight (BW). The treating physician then assessed the severity of CD using the TSUI score (19), while patients rated their CD severity as a percentage of the severity at the onset of BoNT therapy, using a visual analog scale (0–100; 0 = no symptoms of CD; 100 = CD as severe as pre-treatment (PAS)). Patients had been trained to perform this assessment, as they were required to complete it at the end of each injection cycle, just before receiving their next BoNT treatment. Finally, patients were reinjected with BoNT. After the injection, a blood sample was collected and frozen for later analysis of antibody presence during ongoing treatment.

### 2.2 Data collection

Demographic and treatment-related data (sex, age (=age at recruitment), age at onset of CD, DUR CD (=duration of CD), DUR BoNT (=duration of botulinum neurotoxin treatment), BW (=body weight at recruitment), TSUI (=TSUI score at recruitment), PAS (=patient’s assessment of severity of CD in % of the severity at onset of BoNT therapy), and DOSE (=mean dose of the previous 10 BoNT injections)) were either documented on the day of recruitment or extracted from the charts of the patients.

Blood samples were deep-frozen and stored until all samples of all patients had been collected. All samples were then shipped on ice to the BioProof AG laboratory (Munich, Germany). All the samples were stored in one batch so that they could be analyzed using the same ELISA test. Samples of ELISA-positive patients were sent to the Toxicon laboratory (MHH Hannover, Germany) for a confirmation test by means of the MHDA (20).

### 2.3 Assessment of quality of life by means of the CDQ-24

The effects of treatment on QoL were assessed on the day of recruitment using the validated CDQ24 (18). The CDQ24 comprises 24 items designed as a disease-specific QoL instrument to score five different aspects of QoL in CD: stigmatization (sum of questions 7, 8, 9, 10, 18, and 22 = CDQ24-A), emotional wellbeing (sum of questions 11, 12, 13, 14, and 15 = CDQ24-B), pain (sum of questions 4, 5, and 21 = CDQ24-C), activities of daily living (sum of questions 1, 2, 3, 6, 19, and 20 = CDQ24-D), and social/family life (sum of questions 16, 17, 23, 24 = CDQ24-E). Scores for each item range from 0 to 4, representing increasing severity of impairment. The total sum (CDQ24) and the five sub-scores (CDQ24-A–CDQ24-E) were analyzed.

### 2.4 Statistical analysis

**Figure 1:**
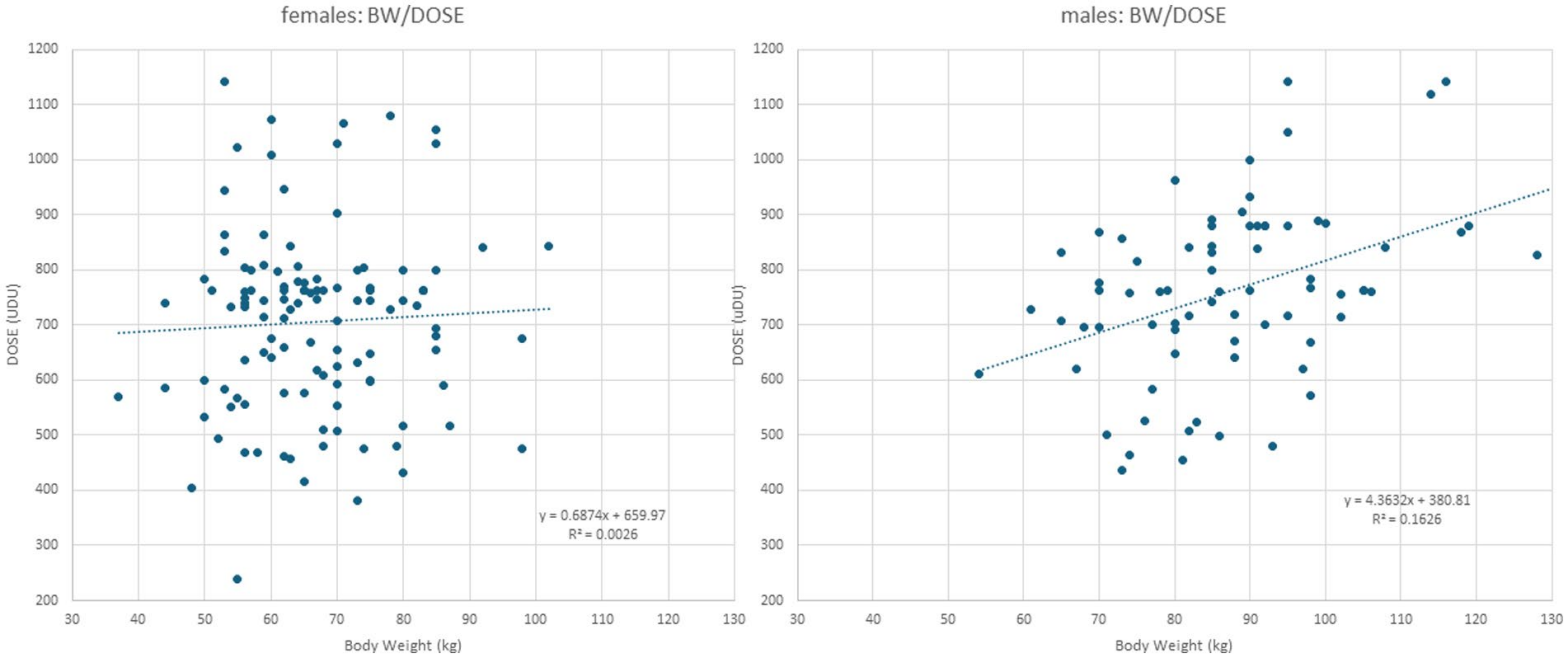
Left part: When DOSE (y-axis) is plotted against BW in female patients, a simple regression line results, indicating that no correlation between BW and DOSE can be detected. Right part: When DOSE (y-axis) is plotted against BW in male patients, a significant (*p* < 0.002) correlation between BW and DOSE is found.

**Figure 2:**
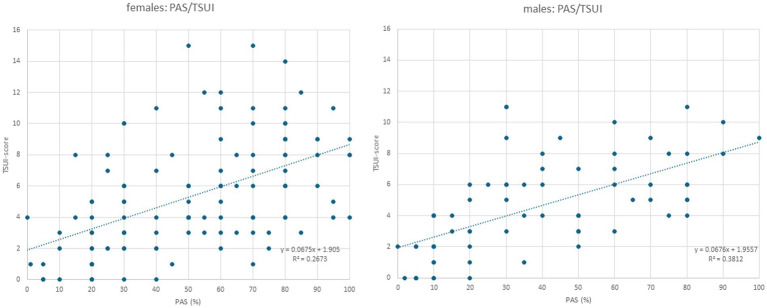
Left part: When the TSUI score (y-axis) is plotted against PAS in female patients, a highly significant (*p* < 0.0001) increasing regression line is found. Right part: When the TSUI score (y-axis) is plotted against PAS in the male patients, a nearly identical regression line with a highly significant correlation (*p* < 0.0001) between TSUI and PAS is found (compared also the small inserts in the left and right parts of this figure).

Non-parametric methods were used to analyze correlations between demographic, treatment-related, and outcome variables. Linear regression models were calculated to visualize relationships and are included in Figures 1, 2. An ANOVA was used to assess differences in variables such as TSUI scores, PAS, and total CDQ-24 scores between the subgroups. Post-hoc pairwise comparisons were performed if significant effects were detected. Cross-correlation matrices explored relationships between body weight (BW), BoNT dose (DOSE), and outcome measures (TSUI, PAS, CDQ24, and CDQ24-A–E), with notable differences between male and female patients being highlighted. To determine the relationship between PAS and TSUI, regression analyses were conducted separately for male and female patients, revealing similar regression slopes and intercepts. To account for the potential confounding effect of BoNT dose on outcome differences, ANCOVA was applied, demonstrating that sex differences in CDQ-24 and PAS persisted even after controlling for dose levels. All statistical analyses were performed using SPSS software, with a significance threshold set at a *p*-value of < 0.05.

## 3 Results

### 3.1 Demographical data of female and male CD patients

Among the 220 patients with idiopathic cervical dystonia (CD), 135 were female (female-to-male ratio: 135/85 = 1.6:1). The mean age of the female patients (62.2 years) was significantly (*p* < 0.02) higher than that of the male patients (58.3 years). The mean duration of disease (DURD) was 17.0 years in the female and 18.6 years in the male patients and did not differ significantly. The mean duration of BoNT therapy was nearly equal in both patient subgroups (female patients: 11.4 years; male patients: 11.9 years; n.s.). The mean age of manifestation of CD (=age of onset of CD; AOS = AGE-DURD) was 43.1 years in the entire cohort. It was increasingly significantly (*p* < 0.001) lower in male (39.7 years) than in the female patients (45.2 years). Mean body weight (BW) was increasingly significantly (*p* < 0.001) lower in the female (67.6 kg) compared to the male patient subgroup (87.8 kg) (for further details, see Table 1).

**Table 1:** Demographics, treatment-related data, and outcomes in female and male patients.

Parameters	Female	Male	Test variable	*p*-value
***n*=**	135	85		
**AGE (years)**	62.2/12.2	58.3/10.9	−2.45	**0.02**
**DUR CD (years)**	17.0/7.60	18.6/10.5	−0.50	0.5
**DUR BoNT (years)**	11.4/5.30	11.9/5.30	0.69	0.49
**Body weight (kg)**	67.6/13.8	87.8/16.0	9.43	**<0.001**
**TSUI**	5.21/3.44	4.64/2.96	−1.30	0.19
**PAS (%)**	51.5/26.7	38.7/27.0	−3.17	**0.002**
**DOSE (uDU)**	708/163	759/153	2.39	**0.02**
**NAB (*n*/%)**	26/19.26	13/15.29	6.30	**0.006**
**CDQ24**	24.3/17.6	18.0/15.5	−2.79	**0.006**
**CDQ24-A**	7.23/6.64	6.22/6.35	−1.12	0.27
**CDQ24-B**	4.98/4.96	2.82/3.63	−3.68	**0.0003**
**CDQ24-C**	4.07/3.31	2.79/3.09	−2.88	**0.004**
**CDQ24-D**	7.15/5.47	5.18/4.45	−2.91	**0.004**
**CDQ24-E**	0.90/1.88	0.95/2.18	0.20	0.84

n = number of patients; AGE = age at recruitment; DUR CD = duration of cervical dystonia; DUR BoNT = duration of botulinum neurotoxin treatment; BW = body weight; TSUI = TSUI score at recruitment; PAS = patient’s assessment of the severity of CD in % of the severity at onset of BoNT therapy; DOSE = mean dose per session during the last 10 BoNT injections; NAB = number and percent of NAB+ patients; CDQ24 = sum of all five sub-scores CDQ24-A–CDQ24-E; CDQ24-A–CDQ24-E = the five different sub-scores of the CDQ24. Bold values indicate statistically significant *p*-values (*p* < 0.05).

### 3.2 Treatment-related data of female and male patients

The majority of the female patients (59.3%; n = 80) had been treated with abobotulinumtoxin type A (aboBoNT/A), 21.5% (n = 29) with onabotulinumtoxin type A (onaBoNT/A), 17.8% (n = 24) with incobotulinumtoxin type A (incoBoNT/A), and only 1% (n = 2) with rimabotulinumtoxin type B (rimaBoNT/B). The distribution of the BoNT preparations used for the treatment of the male patients was similar (aboBoNT/A: n = 59 (69.4%), onaBoNT/A: n = 11 (12.9%), incoBoNT/A: n = 14 (16.0%), rimaBoNT/B: n = 1 (1%)). The chi-squared test did not detect significant differences between the distributions of BoNT preparations (*p* = 0.166). For the sake of comparison, the doses of the different BoNT preparations were transformed into unified dose units (uDU) by leaving onaBoNT/A and incoBoNT/A doses unchanged, by dividing aboBoNT/A doses by a factor of 3.81, and by dividing rimaBoNT/A doses by a factor of 38.1. These conversion ratios have been used previously (11). The mean dose (uDU) was significantly (*p* < 0.02) lower in the female subgroup (female patients: 708 uDU; male patients: 759 uDU). However, the ratio of DOSE/BW was slightly higher in the female subgroup (female patients: 10.47 uDU/kg; male patients: 8.64 uDU/kg) (for further details, see Table 1).

### 3.3 Comparison of the primary outcome measure: the results of the CDQ24

All 220 CD patients completed the CDQ24 questionnaire (the primary outcome measure of the present study), which was used to score five aspects of patients´ quality of life. The total CDQ24 (sum of all 24 sub-scores) was increasingly significantly (*p* < 0.006) lower in male patients (CDQ24: 18.0) compared to female patients (CDQ24: 24.3).

Interestingly, the sub-scores CDQ24-A (stigmatization) and CDQ24-E (aspects of social life and family) did not differ between male and female patients (see Table 1), whereas the sub-scores CDQ24-B (emotional wellbeing), CDQ24-C (pain), and CDQ24-D (interference with daily living) were significantly (*p* < 0.004) higher in female patients (for further details, see Table 1).

### 3.4 Comparison of the secondary outcome measure: the results of PAS

Patients had been trained to assess the remaining severity of CD (PAS) at the end of an injection cycle just before they received their next BoNT injection. Furthermore, this parameter revealed a significant (*p* < 0.002) difference between female and male patients (see Table 1).

### 3.5 Comparison of physicians’ rating of the severity of CD by means of the TSUI score

For all patients and all BoNT injections, the remaining severity of CD was scored by the treating physician at the end of an injection cycle, just before the next BoNT injection was applied. The TSUI score in the female patients was slightly worse (5.21) than that in the male patients (4.64), but the difference was not significant (*p* = 0.19) (see Table 1).

### 3.6 Cross-correlations of demographic, treatment-related, and outcome data

To analyze in more detail whether there are differences in the disease management of female and male patients during BoNT therapy, cross-correlations between all parameters listed in Table 1 (except the NAB data) were performed (Tables 2, 3). The upper parts of Tables 2, 3 show the non-parametric cross-correlation coefficients, and the lower parts show the corresponding *p*-values.

**Table 2:** Cross correlation analysis of the data of the female patients.

**Parameter**	**AGE**	**BW**	**TSUI**	**DUR CD**	**DUR BoNT**	**DOSE**	**PAS**	**CDQ24**
**AGE**	1	0.0850	0.0168	0.4317	0.3104	0.0760	0.0371	0.1811
**BW**	0.3325	1	0.0460	0.0318	0.0727	0.0233	0.0995	-0.0543
**TSUI**	0.8464	0.6006	1	0.0520	0.0237	0.5460	0.5536	0.3978
**DUR CD**	**1.17 × 10** ^**-6**^	0.7369	0.5778	1	0.7029	0.1295	-0.1165	0.1776
**DUR BoNT**	**0.0004**	0.4280	0.7935	**1.01 × 10** ^**-18**^	1	0.1872	0.1486	0.1771
**DOSE**	0.4133	0.8038	**1.61 × 10** ^**-10**^	0.1776	**0.0451**	1	0.2562	0.1618
**PAS**	0.6902	0.2860	**7.93 × 10** ^**-11**^	0.2410	0.1214	**0.0087**	1	0.4884
**CDQ24**	**0.0355**	0.5360	**1.77 × 10** ^**-6**^	0.0554	**0.0491**	**0.0801**	**2.02 × 10** ^**-8**^	1
**CDQ24-A**	**0.0423**	0.4833	**1.54 × 10** ^**-7**^	0.0761	**0.0492**	**0.0260**	**2.55 × 10** ^**-10**^	**5.85 × 10** ^**-40**^
**CDQ24-B**	0.1376	0.3981	0.1253	0.3456	0.4703	0.3847	**0.0045**	**2.07 × 10** ^**-26**^
**CDQ24-C**	0.5966	0.3830	**0.0082**	0.1145	**0.0417**	0.1765	**2.57 × 10** ^**-5**^	**6.44 × 10** ^**-17**^
**CDQ24-D**	**0.0229**	0.3191	**1.44 × 10** ^**-6**^	0.0791	0.0985	0.1134	**1.04 × 10** ^**-5**^	**1.07 × 10** ^**-30**^
**CDQ24-E**	0.1141	0.3130	**0.0301**	0.1453	**0.0479**	0.1115	**0.0265**	**1.05 × 10** ^**-13**^

Cross-correlation analysis of the following parameters: AGE = age at recruitment; BW = body weight; TSUI = TSUI score at recruitment; DUR CD = duration of cervical dystonia; DUR BoNT = duration of botulinum neurotoxin treatment; DOSE = mean dose per session during the last 10 BoNT injections; PAS = patient’s assessment of the severity of CD in % of the severity at onset of BoNT therapy; CDQ24 = sum of all five subscores CDQ24-A–CDQ24-E. The upper part presents correlation coefficients r, and the lower part presents the corresponding *p*-values. Significant correlations are bold. All sub-scores CDQ24-A to CDQ24-E were correlated with each other. Therefore, this part of the cross-correlation matrix was omitted.

**Table 3:** Cross-correlation analysis of the data of male patients.

**parameter**	**AGE**	**BW**	**TSUI**	**DUR CD**	**DUR BoNT**	**DOSE**	**PAS**	**CDQ24**
**AGE**	1	0.0061	0.3302	0.5630	0.3042	0.1250	0.2008	0.1411
**BW**	0.9565	1	0.0913	0.0458	0.0152	0.3578	0.2604	0.0091
**TSUI**	**0.0020**	0.4149	1	0.2015	0.0184	0.4864	0.6683	0.2935
**DUR CD**	**9.84 × 10** ^**-8**^	0.6984	0.0789	1	0.6038	0.0698	0.0456	-0.0820
**DUR BoNT**	**0.0072**	0.8978	0.8737	**6.12 × 10** ^**-9**^	1	0.0725	0.1412	0.2140
**DOSE**	0.2818	**0.0016**	**8.40 × 10** ^**-6**^	0.5574	0.5424	1	0.3556	0.2538
**PAS**	0.0884	**0.0272**	**1.04 × 10** ^**-10**^	0.7160	0.2582	**0.0031**	1	0.5160
**CDQ24**	0.1978	0.9354	**0.0064**	0.4781	0.0617	**0.0269**	**2.98 × 10** ^**-6**^	1
**CDQ24-A**	0.3969	0.9219	**0.0488**	0.5845	0.1076	0.1242	**0.0010**	**1.95 × 10** ^**-26**^
**CDQ24-B**	0.1043	0.3654	0.3844	0.3020	0.1737	0.4298	**0.0152**	**2.46 × 10** ^**-18**^
**CDQ24-C**	0.7682	0.7409	**0.0155**	0.4917	0.5823	0.1114	**0.0001**	**1.01 × 10** ^**-8**^
**CDQ24-D**	0.3133	0.5042	**0.0013**	0.5965	**0.0352**	**0.0255**	**8.44 × 10** ^**-6**^	**6.55 × 10** ^**-27**^
**CDQ24-E**	0.0779	0.6294	0.3062	0.4495	0.1919	0.1261	0.0558	**1.66 × 10** ^**-11**^

Cross-correlation analysis of the following parameters: AGE = age at recruitment; BW = body weight; TSUI = TSUI score at recruitment; DUR CD = duration of cervical dystonia; DUR BoNT = duration of botulinum neurotoxin treatment; DOSE = mean dose per session during the last 10 BoNT injections; PAS = patient’s assessment of the severity of CD in % of the severity at onset of BoNT therapy; CDQ24 = sum of all five subscores CDQ24-A – CDQ24-E. The upper part presents correlation coefficients r, and the lower part the corresponding *p*-values. Significant correlations are bold. With two exceptions, all sub-scores CDQ24-A to CDQ24-E were correlated with each other. Therefore, this part of the cross-correlation matrix was omitted.

In female patients, body weight (BW) did not correlate with any parameter (Table 2; for the presentation of the individual data, see Figure 1, left part). This was different in male patients (Table 3). BW of the male CD patients revealed a significant (*p* < 0.002) correlation with DOSE (for the presentation of individual data, see Figure 1, right part) as well as with PAS (*p* < 0.03).

All three outcome measures revealed significant cross-correlations with each other in the female (Table 2) and male (Table 3) patients. The correlation between patients´ self-assessment of CD severity (PAS) and physicians´ ratings of the remaining severity of CD using the TSUI score was nearly identical between the female and the male patients. This is shown in Figure 2. The regression line between TSUI (y-axis) and PAS (x-axis) had nearly the same slope and intercept for the female and the male patients (female patients: TSUI = 0.0675*PAS + 1.905; r = 0.5170; *p* < 0.001 (Figure 2, left part); male patients: TSUI = 0.0676*PAS + 1.956; r = 0.6174; *p* < 0.001 (Figure 2, right part)).

DOSE was significantly (*p* < 0.03) correlated with all three outcome measures (CDQ24, PAS, TSUI) in both the female and the male subgroups (compared to Tables 2, 3).

Since CDQ24 was correlated with dose in female but not in male patients, an ANCOVA was used to demonstrate that the significant difference between sex groups persisted for CDQ24 and PAS even when the influence of dose was controlled (see Table 4), or that the influence on outcome of SEX was much stronger than DOSE.

**Table 4:** Comparison of influence on outcome of SEX compared to DOSE.

**Parameter**	**test-variable**	**p< (SEX)**	**p<(DOSE)**
**PAS**	6,530	**0.0115**	0.945
**CDQ24**	8.890	**0.0033**	0.807
**CDQ24-A**	1.160	0.282	0.591
**CDQ24-B**	15.74	**<0.001**	0.617
**CDQ24-C**	10.78	**0.0012**	0.979
**CDQ24-D**	6.990	**0.009**	0.632
**CDQ24-E**	0.920	0.338	0.704

Comparison of the influence of SEX and DOSE on PAS and CDQ24 and its sub-scores CDQ24 A–E. Beyond the highly significant influence of SEX on PAS, CDQ24, and CDQ24-B to CDQ24-D, no further influence of DOSE could be detected. Bold values indicate statistically significant *p*-values (*p* < 0.05).

### 3.7 Comparison of NAB formation in female and male CD patients

In all 220 CD patients, an ELISA test was performed to analyze the presence of NABs. NABs were detected in 26 (= 19.3%) of the female patients. In male patients—who had been treated with BoNT for at least as long as the female patients, and received higher doses less frequently—NABs were detected in 13 patients (15.3%, *p* < 0.006).

Table 5 compares the mean values of the four parameters—DOSE, CDQ24, PAS, and TSUI—between NAB+ and NAB− male patients (upper part) and between NAB+ and NAB− female patients (middle part). The dose had been increased more in the female NAB+ patients than in the male NAB+ patients, resulting in better CDQ 24 and PAS scores in the NAB+ compared to the NAB− patients (see lower part of Table 5). This finding underlines the necessity of sufficiently high dosing. The difference in PAS between male NAB+ patients and NAB− patients was +6.8, whereas the difference between female NAB+ patients and NAB− patients was −1.9. This difference between female and male patients was significant (*p* < 0.02).

**Table 5:** Comparison of the difference between NAB+ and NAB− patients.

**subgroup**	*n*=	**TSUI**	**PAS**	**DOSE**	**CDQ24**
**M NAB+**	13	5.92	44.5	821	20.1
**M NAB-**	72	4.40	37.7	747	17.6
test-variable		3.482	3.116	3.391	2.291
**p-value**		**0.001**	**0.002**	**0.001**	**0.024**
**F NAB+**	26	6.15	50.0	794	23.7
**F NAB-**	109	4.98	51.9	686	24.5
test-variable		1.941	0.630	3.584	-0.531
**p-value**		0.056	0.530	**0.001**	0.597
**M DIFF NAB+/-**		1.52	6.80	74	2.5
**F DIFF NAB+/-**		1.17	-1.90	108	-0.80
test-variable		-0.470	-2.338	-1.774	0.914
**p-value**		0.639	**0.020**	0.362	0.078

Comparison of TSUI, PAS, DOSE, and CDQ24 in male (M) patients with (NAB+) and without (NAB-) antibody formation (upper part of this table). All four parameters revealed a significant difference between the two antibody subgroups. In the female patients (middle part of this table), only DOSE showed a significant difference between NAB+ and NAB− female patients. When the differences of the four parameters (TSUI, PAS, DOSE, and CDQ24) between NAB+ and NAB− patients in the female and male patient subgroups (lower part of this table) were compared, PAS was significantly (*p* < 0.02) different. Bold values indicate statistically significant *p*-values (*p* < 0.05).

## 4 Discussion

### 4.1 Typical demographic data of the present cohort of CD patients

The patients in the present study represent a typical cohort of Caucasian patients with idiopathic cervical dystonia. The mean age of the onset of symptoms was 43.1 years, which is very close to the 42.0 years reported in (1). Furthermore, the female-to-male ratio of 1.6:1 fits the mean female-to-male ratio of 1.7:1 across 16 epidemiological studies reported in (1). Even the significantly earlier age of CD manifestation in male patients compared to female patients has been observed in several previous epidemiological studies (21–24).

### 4.2 Differences in outcome between female and male CD patients

Three different outcome measures were analyzed in the present study: the CDQ24 questionnaire (18), the patient’s assessment of the remaining severity of CD in percent of the severity of CD at the onset of BoNT therapy (PAS), and the physicians’ scoring of the remaining severity of CD at the end of an injection cycle using the TSUI score (TSUI (19)). All three parameters were increasingly significantly correlated with each other (see Tables 2, 3). CDQ24 and PAS were significantly worse in female patients than in male patients, although the mean duration of continuous treatment was approximately 11.5 years without a difference between the patient subgroups. No significant difference was found in the TSUI score.

Interestingly, PAS (patient’s assessment of the efficacy of BoNT treatment, scoring the remaining severity of CD in percent of the initial severity of CD at the onset of BoNT therapy) was correlated with TSUI (physicians’ assessment of the remaining severity of CD). Highly significant (*p* < 0.001) correlations and nearly identical regression lines between PAS and TSUI were found for female and male patients (see inserts in the left and right part of Figure 2). Therefore, the assessment of the clinical appearance of CD did not differ between female and male patients.

This is in full agreement with the fact that aspects of stigmatization scored by the CDQ24-A sub-score did not differ between female and male patients. Furthermore, the implications of CD on social life or family (CDQ24-E) did not show significant differences between female and male patients. The differences in outcome primarily result from aspects of the body scheme and self-awareness, including pain.

### 4.3 Differences in the treatment of female and male CD patients

The most striking difference between female and male patients was body weight (BW), which was to be expected. In the entire cohort, BW did not correlate with any other parameter analyzed in the present study. At our institution, the treatment strategy for patients with CD is to adapt the dose based on the severity of CD rather than body weight. As a result, highly significant correlations were observed between DOSE and TSUI, PAS, and CDQ24.

This is different from the treatment of patients with spasticity. In the BoNT treatment of spasticity in children with infantile cerebral palsy (ICP), dosing according to BW is a standard procedure (15, 17). Nevertheless, there has been an ongoing debate since the first studies on BoNT treatment of ICP (25, 26) on whether patients should be treated with low or high doses per BW. Both the efficacy of treatment and severe and systemic side effects appear to increase with dose per BW (17, 27). In CD, no such recommendations exist.

A closer look at the data from the present study reveals that female and male patients were treated differently. In the male patients, a significant increase in dose with body weight was observed (Figure 1, right part), whereas in the female patients, no dose adaptation to BW was observed (Figure 1, left part). In women with a high BW, the BoNT dose had not been increased, resulting in a significantly lower mean BoNT dose and, therewith, lower mean TSUI and lower CDQ24 scores. This discrepancy had occurred unconsciously, and the treating physicians were unaware of the different treatment strategies they had applied for the treatment of female and male CD patients over the years. Female patients, who were treated without any dose adaptation to BW, rated their outcomes as worse than male patients who received doses adapted to BW.

DOSE was significantly higher in female patients who tested positive for NAB (NAB+ patients) than in NAB− patients (see Table 5). It is highly likely that, in these patients, a pSTF had already developed over the years so that the dose per session had been increased more and more up to 794 uDU, which is close to the 821 uDU in male NAB+ patients (Table 5). As a result, CDQ24 and PAS were better in female NAB+ compared to female NAB− patients. This was not the case in male patients (see Table 5). This finding demonstrates that, in the present cohort of still-responding CD patients, each dose increase leads to further improvement, indicating that female patients, especially those with a higher BW, had potentially been underdosed in the present cohort.

### 4.4 The difference in treatment dose does not completely explain the difference in outcome

To control the influence of dose, an ANCOVA was calculated with dose as a covariate. Even when differences in dose are taken into account, PAS and CDQ24-B, CDQ24-C, and CDQ-D remain significantly (*p* < 0.004) different between female and male patients (see Table 4). The most significant difference was observed in the aspects of emotional wellbeing (CDQ24-B).

This indicates that female patients with CD may suffer more from disturbances in their self-image or inner representation of their own body than male patients with CD. Therefore, future studies should explore whether a simple increase in dose will not completely compensate for the difference in outcome between male and female patients.

### 4.5 Difference in NAB formation cannot be excluded

Although the mean dose per session of BoNT was lower in female patients than in male patients, the frequency of NAB induction was significantly higher in the female subgroup. This finding is in line with the well-established fact that autoimmune diseases are more common in female than in male patients (28) and autoimmune diseases in female patients are more often associated with additional autoimmune conditions compared to male patients (29). A more active immune system in female patients can therefore account for the higher prevalence of NABs in female than in male patients in the present study.

However, theoretically, other factors, such as a larger diffusion of BoNT through muscle membranes in female than in male patients, can provide a possible explanation. Compared to the difference in autoimmunity, these factors are less well analyzed.

Nevertheless, we recommend optimizing the injection scheme and interval before increasing the BoNT dose and choosing the BoNT preparation with the lowest antigenicity, regardless of the patient’s sex.

## 5 Conclusion

The present study demonstrates that the outcome of BoNT therapy is rated worse by female CD patients compared to male CD patients, despite minimal differences in the clinically apparent symptoms of CD. Female patients with a higher body weight were likely underdosed. However, differences in dosing appear to play a less significant role than aspects of self-awareness. We therefore recommend conducting further studies and a meta-analysis of existing data to analyze the differential outcomes in female and male patients during BoNT therapy in greater detail. We also recommend further studies on NAB formation during BoNT therapy and whether the difference in the prevalence of NABs in the present study can be confirmed by other centers.

## 6 Strengths and limitations of the study

The strength of the present study is that it demonstrates a difference in treatment outcomes between female and male patients during BoNT long-term therapy, particularly in terms of quality of life and patients´ assessment of treatment efficacy. A key limitation of the study is that it is a monocentric study and relies primarily on the CDQ24 as the only quality-of-life measurement instrument tool, as well as on the relatively simple TSUI score to assess the severity of CD. Therefore, a prospective study is recommended—one that more precisely controls for BoNT dose and CD severity and evaluates the differential influence of BoNT therapy on the quality of life of female and male patients using additional quality-of-life instruments in addition to the CDQ24. Such a study should also explore the potential influence of psychiatric factors such as depression and prospectively analyze the incidence and prevalence of both NAB formation and autoimmune diseases.

## Data Availability

The raw data supporting the conclusions of this article will be made available by the authors, without undue reservation.
